# 1,3-Diphenyl-4,5-dihydro-1*H*-pyrazol-5-one

**DOI:** 10.1107/S1600536812009567

**Published:** 2012-03-10

**Authors:** Thomas C. Baddeley, Solange M. S. V. Wardell, Edward R. T. Tiekink, James L. Wardell

**Affiliations:** a Department of Chemistry, University of Aberdeen, Meston Walk, Old Aberdeen AB24 3UE, Scotland; b CHEMSOL, 1 Harcourt Road, Aberdeen, AB15 5NY, Scotland; c Department of Chemistry, University of Malaya, 50603 Kuala Lumpur, Malaysia; d Centro de Desenvolvimento Tecnológico em Saúde (CDTS), Fundação Oswaldo Cruz (FIOCRUZ), Casa Amarela, Campus de Manguinhos, Av. Brasil 4365, 21040-900 Rio de Janeiro, RJ, Brazil

## Abstract

In the title pyrazolone derivative, C_15_H_12_N_2_O, the five-membered ring is approximately planar (r.m.s. deviation = 0.018 Å), and the N- and C-bound benzene rings are inclined to this plane [dihedral angles = 21.45 (10) and 6.96 (10)°, respectively] and form a dihedral angle of 20.42 (10)° with each other. Supra­molecular layers are formed in the crystal structure *via* C—H⋯O and C—H⋯N inter­actions, and these are assembled into double layers by C—H⋯π and π–π inter­actions between the pyrazole and C-bound benzene rings [ring centroid–centroid distance = 3.6476 (12) Å]. The double layers stack along the *a* axis being connected by π–π inter­actions between the N- and C-bound benzene rings [ring centroid–centroid distance = 3.7718 (12) Å].

## Related literature

For the therapeutic importance of pyrazoles, see: Sil *et al.* (2005 ▶); Haddad *et al.* (2004 ▶). For their diverse pharmacological activities, see: Bekhit *et al.* (2012 ▶); Castagnolo *et al.* (2008 ▶); Ramajayam *et al.* (2010 ▶). For background to the synthesis, see: Nef (1891 ▶); Katritzky *et al.* (1997 ▶); Wardell *et al.* (2007 ▶); de Lima *et al.* (2010 ▶). For evaluation of tautomeric forms using NMR MO calculations and crystallography, see: Feeney *et al.* (1970 ▶); Hawkes *et al.* (1977 ▶); Freyer *et al.* (1983 ▶); Dardonville *et al.* (1998 ▶); Kleinpeter & Koch (2001 ▶); Bechtel *et al.* (1973*a* ▶,*b* ▶); Chmutova *et al.* (2001 ▶); Wardell *et al.* (2007 ▶); Gallardo *et al.* (2009 ▶); Ding & Zhao (2010 ▶). For a previous synthesis, see: Kimata *et al.* (2007 ▶). For a recently reported structure, see: Wardell *et al.* (2012 ▶).
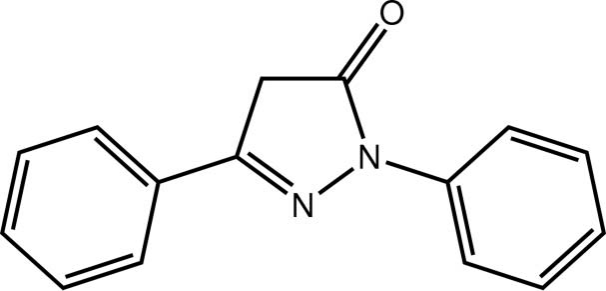


## Experimental

### 

#### Crystal data

C_15_H_12_N_2_O
*M* *_r_* = 236.27
Monoclinic, 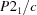

*a* = 11.1823 (3) Å
*b* = 11.7503 (4) Å
*c* = 9.6443 (2) Å
β = 113.998 (2)°
*V* = 1157.68 (6) Å^3^
*Z* = 4
Mo *K*α radiation
μ = 0.09 mm^−1^
*T* = 120 K
0.34 × 0.10 × 0.08 mm

#### Data collection

Rigaku Saturn724+ diffractometer
Absorption correction: multi-scan (*SADABS*; Sheldrick, 2007 ▶) *T*_min_ = 0.790, *T*_max_ = 1.000
12058 measured reflections
2024 independent reflections
1829 reflections with *I* > 2σ(*I*)
*R*_int_ = 0.042

#### Refinement

*R*[*F* ^2^ > 2σ(*F* ^2^)] = 0.048
*wR*(*F* ^2^) = 0.127
*S* = 1.07
2024 reflections
163 parameters
H-atom parameters constrained
Δρ_max_ = 0.74 e Å^−3^
Δρ_min_ = −0.20 e Å^−3^

### 

Data collection: *COLLECT* (Hooft, 1998 ▶); cell refinement: *DENZO* (Otwinowski & Minor, 1997 ▶) and *COLLECT*; data reduction: *DENZO* and *COLLECT*; program(s) used to solve structure: *SHELXS97* (Sheldrick, 2008 ▶); program(s) used to refine structure: *SHELXL97* (Sheldrick, 2008 ▶); molecular graphics: *ORTEP-3* (Farrugia, 1997 ▶) and *DIAMOND* (Brandenburg, 2006 ▶); software used to prepare material for publication: *publCIF* (Westrip, 2010 ▶).

## Supplementary Material

Crystal structure: contains datablock(s) global, I. DOI: 10.1107/S1600536812009567/hg5186sup1.cif

Structure factors: contains datablock(s) I. DOI: 10.1107/S1600536812009567/hg5186Isup2.hkl

Supplementary material file. DOI: 10.1107/S1600536812009567/hg5186Isup3.cml

Additional supplementary materials:  crystallographic information; 3D view; checkCIF report

## Figures and Tables

**Table 1:** Hydrogen-bond geometry (Å, °) *Cg*1 is the centroid of the C10–C15 ring.

*D*—H⋯*A*	*D*—H	H⋯*A*	*D*⋯*A*	*D*—H⋯*A*
C8—H8*A*⋯O1^i^	0.99	2.36	3.279 (2)	154
C12—H12⋯N2^ii^	0.95	2.61	3.527 (2)	163
C8—H8*B*⋯*Cg*1^iii^	0.99	2.69	3.437 (2)	132

Symmetry codes: (i) 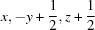; (ii) 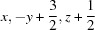; (iii) 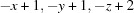.

**Fig. 1.**
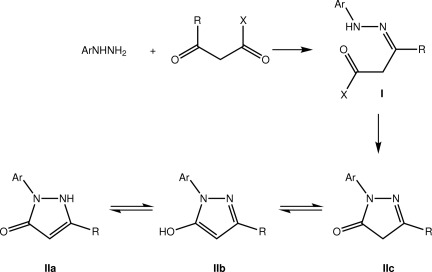
Reaction scheme.

**Fig. 2.**
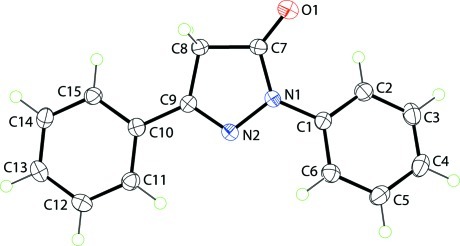
The molecular structure of (I) showing the atom-labelling scheme and displacement ellipsoids at the 50% probability level.

**Fig. 3.**
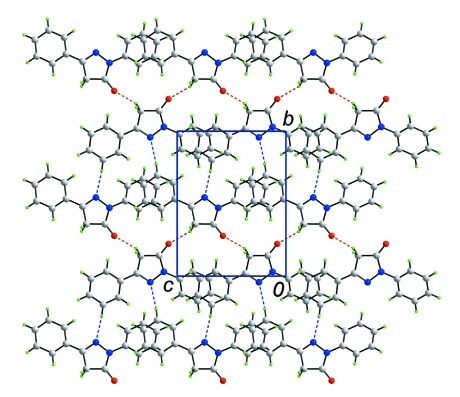
Supramolecular layer in (I) sustained by C—H···O and C—H···N interactions shown as orange and blue dashed lines, respectively.

**Fig. 4.**
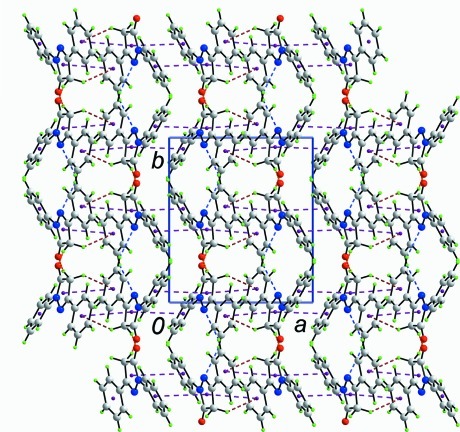
A view in projection down the c axis of the crystal packing in (I). The C—H···O, C—H···N, C—H···π and π–π interactions shown as orange, blue, brown and purple dashed lines, respectively.

## supplementary crystallographic information

### Comment

Pyrazoles are key structures in numerous compounds of therapeutic importance (Sil *et al.*, 2005, Haddad *et al.*, 2004). Compounds containing this ring system are known to display diverse pharmacological activities, for example as anti-malarial agents (Bekhit *et al.*, 2012), anti-tuberculosis agents (Castagnolo *et al.*, 2008), and as SARS-coronavirus protease inhibitors (Ramajayam *et al.*, 2010).

A general route to pyrazole derivatives involves reaction of an arylhydrazine, ArNHNH_2_, with a β-dicarbonyl compound, *R*/COCH_2_COX.. This reaction provides initially a hydrazone derivative, RNHN=CR/CH_2_COX, I (Fig. 1), which can be isolated but which readily undergoes cyclization to a pyrazone derivative, II (Fig.1), (Nef, 1891; Katritzky *et al.*, 1997; Wardell *et al.*, 2007; de Lima *et al.*, 2010). Equilibrium involving tautomers of II in solution have been variously studied using NMR and IR spectroscopy and *ab initio* calculations. (Feeney *et al.*, 1970; Hawkes *et al.*, 1977; Freyer *et al.*, 1983; Dardonville *et al.*, 1998; Kleinpeter & Koch, 2001). Crystal structures of various pyrazone compounds of forms IIa, IIb and IIc have been reported (see for example, Bechtel *et al.*, 1973*a*; Bechtel *et al.*, 1973*b*; Chmutova *et al.*, 2001; Wardell *et al.*, 2007; Gallardo *et al.*, 2009; Ding & Zhao, 2010). In continuation of recent studies (Wardell *et al.*, 2012), herein, the isolation the title compound [*i.e*. form IIc (Fig. 1)] from the reaction between PhNHNH_2_ and PhCOCH_2_CO_2_Et in EtOH is described as is its crystal structure. The same tautomer was also isolated in the reaction between PhNHNH_2_ and PhCOCH_2_CONHPh in EtOH.

In the title compound, Fig. 2, crystallography proves the IIc tautomer in the solid-state. The pyrazole ring is planar with a r.m.s. deviation for the fitted atoms of 0.018 Å; the maximum deviations from this plane are 0.015 (1) Å (for the N1 atom) and -0.015 (1) Å (C8). The N– and C-bound benzene rings are inclined to this plane forming dihedral angles of 21.45 (10) and 6.96 (10)°, respectively; the dihedral angle between the benzene rings is 20.42 (10)° consistent with a non-planar molecule.

In the crystal structure, supramolecular layers are formed in the *bc* plane through C—H···O and C—H···N interactions, Fig. 3 and Table 1. Large 21-membered {···NC_4_H···N_2_CO···HC_2_O···HC_5_H} synthons are formed through these interactions. Layers are connected into double layers by C—H···π, Table 1, and π–π interactions formed between the pyrazole and C-bound benzene rings [ring centroid···centroid distance = 3.6476 (12) Å, angle of inclination of 6.96 (10)° for symmetry operation 1 - *x*, 1 - *y*, 2 - *z*]. The double layers stack along the *a* axis being connected by π–π interactions between the N– and C-bound benzene rings [ring centroid···centroid distance = 3.7718 (12) Å, angle of inclination of 21.45 (10)° for symmetry operation -*x*, 1 - *y*, 1 - *z*].

### Experimental

A solution of PhNHNH_2_ (1 mmol) and PhCOCH_2_CO_2_Et (1 mmol) in EtOH (15 ml) was refluxed for 1 h. The reaction mixture was maintained at room temperature and crystals of the titled compound were collected after 2 days, *M*.pt: 408–410 K: lit. value 409–411 K (Kimata *et al.*, 2007). IR and NMR spectra are in agreement with published data (Castagnolo *et al.*, 2008). MS—MS (*M*+H = 237): 209, 195, 106, 91.

### Refinement

The C-bound H atoms were geometrically placed (C—H = 0.95–0.99 Å) and refined as riding with *U*_iso_(H) = 1.2*U*_eq_(C). Owing to poor agreement one reflection, *i.e*. (3 1 1), was removed from the final cycles of refinement. The maximum and minimum residual electron density peaks of 0.74 and 0.20 e Å^-3^, respectively, were located 1.02 Å and 0.58 Å from the H8a and O1 atoms, respectively.

### Crystal data

**Table:**

C_15_H_12_N_2_O	*F*(000) = 496
*M**_r_* = 236.27	*D*_x_ = 1.356 Mg m^−^^3^
Monoclinic, *P*2_1_/*c*	Mo *K*α radiation, λ = 0.71073 Å
Hall symbol: -P 2ybc	Cell parameters from 9201 reflections
*a* = 11.1823 (3) Å	θ = 2.9–27.5°
*b* = 11.7503 (4) Å	µ = 0.09 mm^−^^1^
*c* = 9.6443 (2) Å	*T* = 120 K
β = 113.998 (2)°	Rod, light-yellow
*V* = 1157.68 (6) Å^3^	0.34 × 0.10 × 0.08 mm
*Z* = 4	

### Data collection

**Table:**

Rigaku Saturn724+ diffractometer	2024 independent reflections
Radiation source: Rotating Anode	1829 reflections with *I* > 2σ(*I*)
Confocal monochromator	*R*_int_ = 0.042
Detector resolution: 28.5714 pixels mm^-1^	θ_max_ = 25.0°, θ_min_ = 2.9°
profile data from ω–scans	*h* = −13→13
Absorption correction: multi-scan (*SADABS*; Sheldrick, 2007)	*k* = −13→12
*T*_min_ = 0.790, *T*_max_ = 1.000	*l* = −11→11
12058 measured reflections	

### Refinement

**Table:**

Refinement on *F*^2^	Primary atom site location: structure-invariant direct methods
Least-squares matrix: full	Secondary atom site location: difference Fourier map
*R*[*F*^2^ > 2σ(*F*^2^)] = 0.048	Hydrogen site location: inferred from neighbouring sites
*wR*(*F*^2^) = 0.127	H-atom parameters constrained
*S* = 1.07	*w* = 1/[σ^2^(*F*_o_^2^) + (0.0598*P*)^2^ + 0.8279*P*] where *P* = (*F*_o_^2^ + 2*F*_c_^2^)/3
2024 reflections	(Δ/σ)_max_ < 0.001
163 parameters	Δρ_max_ = 0.74 e Å^−^^3^
0 restraints	Δρ_min_ = −0.20 e Å^−^^3^

### Special details

**Table:**

Geometry. All s.u.'s (except the s.u. in the dihedral angle between two l.s. planes) are estimated using the full covariance matrix. The cell s.u.'s are taken into account individually in the estimation of s.u.'s in distances, angles and torsion angles; correlations between s.u.'s in cell parameters are only used when they are defined by crystal symmetry. An approximate (isotropic) treatment of cell s.u.'s is used for estimating s.u.'s involving l.s. planes.
Refinement. Refinement of *F*^2^ against ALL reflections. The weighted *R*-factor *wR* and goodness of fit *S* are based on *F*^2^, conventional *R*-factors *R* are based on *F*, with *F* set to zero for negative *F*^2^. The threshold expression of *F*^2^ > 2σ(*F*^2^) is used only for calculating *R*-factors(gt) *etc*. and is not relevant to the choice of reflections for refinement. *R*-factors based on *F*^2^ are statistically about twice as large as those based on *F*, and *R*- factors based on ALL data will be even larger.

### Fractional atomic coordinates and isotropic or equivalent isotropic displacement parameters (Å^2^)

**Table:**

	*x*	*y*	*z*	*U*_iso_*/*U*_eq_	
O1	0.22694 (13)	0.27778 (11)	0.57957 (14)	0.0312 (4)	
N1	0.21383 (13)	0.47099 (12)	0.62331 (15)	0.0198 (4)	
N2	0.24220 (13)	0.54258 (12)	0.74837 (15)	0.0196 (3)	
C1	0.15405 (16)	0.51898 (14)	0.47568 (18)	0.0195 (4)	
C2	0.16304 (18)	0.46485 (15)	0.35195 (19)	0.0247 (4)	
H2	0.2084	0.3946	0.3650	0.030*	
C3	0.1051 (2)	0.51443 (17)	0.2092 (2)	0.0302 (5)	
H3	0.1101	0.4771	0.1243	0.036*	
C4	0.03995 (18)	0.61747 (16)	0.1888 (2)	0.0292 (5)	
H4	0.0002	0.6507	0.0907	0.035*	
C5	0.03356 (17)	0.67148 (16)	0.3133 (2)	0.0272 (4)	
H5	−0.0094	0.7429	0.3004	0.033*	
C6	0.08893 (17)	0.62284 (15)	0.4563 (2)	0.0232 (4)	
H6	0.0826	0.6600	0.5405	0.028*	
C7	0.24708 (16)	0.35860 (14)	0.66481 (19)	0.0200 (4)	
C8	0.30948 (17)	0.35961 (14)	0.83575 (18)	0.0202 (4)	
H8A	0.2618	0.3096	0.8787	0.024*	
H8B	0.4023	0.3357	0.8752	0.024*	
C9	0.29726 (16)	0.48130 (14)	0.86983 (18)	0.0187 (4)	
C10	0.34323 (16)	0.53258 (14)	1.02117 (18)	0.0204 (4)	
C11	0.31799 (17)	0.64722 (15)	1.0390 (2)	0.0231 (4)	
H11	0.2693	0.6921	0.9523	0.028*	
C12	0.36371 (18)	0.69541 (16)	1.1822 (2)	0.0259 (4)	
H12	0.3452	0.7729	1.1937	0.031*	
C13	0.43659 (18)	0.63072 (16)	1.3092 (2)	0.0271 (4)	
H13	0.4688	0.6642	1.4073	0.033*	
C14	0.46240 (18)	0.51717 (16)	1.2925 (2)	0.0260 (4)	
H14	0.5127	0.4731	1.3794	0.031*	
C15	0.41501 (17)	0.46785 (15)	1.14959 (19)	0.0225 (4)	
H15	0.4315	0.3897	1.1390	0.027*	

### Atomic displacement parameters (Å^2^)

**Table:**

	*U*^11^	*U*^22^	*U*^33^	*U*^12^	*U*^13^	*U*^23^
O1	0.0396 (8)	0.0244 (7)	0.0291 (7)	0.0014 (6)	0.0134 (6)	−0.0030 (6)
N1	0.0215 (7)	0.0188 (8)	0.0177 (7)	−0.0005 (6)	0.0064 (6)	−0.0021 (5)
N2	0.0202 (7)	0.0191 (8)	0.0195 (7)	−0.0009 (6)	0.0079 (6)	−0.0019 (5)
C1	0.0169 (8)	0.0201 (9)	0.0201 (9)	−0.0037 (6)	0.0061 (7)	0.0006 (7)
C2	0.0271 (9)	0.0236 (9)	0.0244 (9)	−0.0011 (7)	0.0113 (7)	0.0003 (7)
C3	0.0357 (11)	0.0335 (11)	0.0214 (9)	−0.0065 (8)	0.0117 (8)	−0.0026 (8)
C4	0.0280 (10)	0.0302 (10)	0.0225 (9)	−0.0072 (8)	0.0031 (8)	0.0067 (7)
C5	0.0235 (9)	0.0233 (9)	0.0294 (10)	−0.0014 (7)	0.0053 (7)	0.0042 (7)
C6	0.0216 (9)	0.0230 (9)	0.0243 (9)	−0.0013 (7)	0.0086 (7)	−0.0014 (7)
C7	0.0217 (9)	0.0170 (8)	0.0221 (9)	−0.0005 (7)	0.0099 (7)	−0.0007 (7)
C8	0.0231 (9)	0.0174 (9)	0.0207 (8)	0.0006 (7)	0.0094 (7)	0.0010 (7)
C9	0.0170 (8)	0.0201 (9)	0.0195 (8)	0.0000 (6)	0.0079 (7)	0.0015 (7)
C10	0.0191 (8)	0.0230 (9)	0.0214 (9)	−0.0013 (7)	0.0106 (7)	0.0000 (7)
C11	0.0214 (9)	0.0241 (9)	0.0233 (9)	0.0026 (7)	0.0086 (7)	0.0018 (7)
C12	0.0272 (9)	0.0235 (9)	0.0294 (10)	0.0012 (7)	0.0140 (8)	−0.0040 (7)
C13	0.0286 (10)	0.0316 (11)	0.0217 (9)	−0.0024 (8)	0.0108 (8)	−0.0062 (7)
C14	0.0273 (10)	0.0305 (10)	0.0199 (9)	0.0018 (8)	0.0093 (7)	0.0035 (7)
C15	0.0257 (9)	0.0205 (9)	0.0232 (9)	0.0012 (7)	0.0120 (7)	0.0016 (7)

### Geometric parameters (Å, º)

**Table:**

O1—C7	1.216 (2)	C7—C8	1.506 (2)
N1—C7	1.386 (2)	C8—C9	1.486 (2)
N1—N2	1.3970 (19)	C8—H8A	0.9900
N1—C1	1.421 (2)	C8—H8B	0.9900
N2—C9	1.297 (2)	C9—C10	1.465 (2)
C1—C2	1.391 (2)	C10—C15	1.396 (2)
C1—C6	1.394 (2)	C10—C11	1.401 (2)
C2—C3	1.389 (3)	C11—C12	1.384 (2)
C2—H2	0.9500	C11—H11	0.9500
C3—C4	1.385 (3)	C12—C13	1.389 (3)
C3—H3	0.9500	C12—H12	0.9500
C4—C5	1.386 (3)	C13—C14	1.388 (3)
C4—H4	0.9500	C13—H13	0.9500
C5—C6	1.384 (2)	C14—C15	1.387 (2)
C5—H5	0.9500	C14—H14	0.9500
C6—H6	0.9500	C15—H15	0.9500
			
C7—N1—N2	112.61 (13)	C9—C8—H8A	111.4
C7—N1—C1	128.99 (14)	C7—C8—H8A	111.4
N2—N1—C1	118.38 (14)	C9—C8—H8B	111.4
C9—N2—N1	107.64 (14)	C7—C8—H8B	111.4
C2—C1—C6	120.06 (16)	H8A—C8—H8B	109.3
C2—C1—N1	120.63 (15)	N2—C9—C10	121.10 (15)
C6—C1—N1	119.29 (15)	N2—C9—C8	112.77 (14)
C3—C2—C1	119.40 (17)	C10—C9—C8	126.12 (15)
C3—C2—H2	120.3	C15—C10—C11	119.15 (15)
C1—C2—H2	120.3	C15—C10—C9	120.12 (16)
C4—C3—C2	120.92 (17)	C11—C10—C9	120.72 (15)
C4—C3—H3	119.5	C12—C11—C10	120.30 (16)
C2—C3—H3	119.5	C12—C11—H11	119.8
C3—C4—C5	119.12 (16)	C10—C11—H11	119.8
C3—C4—H4	120.4	C11—C12—C13	120.13 (17)
C5—C4—H4	120.4	C11—C12—H12	119.9
C6—C5—C4	120.94 (17)	C13—C12—H12	119.9
C6—C5—H5	119.5	C14—C13—C12	119.95 (16)
C4—C5—H5	119.5	C14—C13—H13	120.0
C5—C6—C1	119.52 (17)	C12—C13—H13	120.0
C5—C6—H6	120.2	C13—C14—C15	120.21 (16)
C1—C6—H6	120.2	C13—C14—H14	119.9
O1—C7—N1	126.50 (15)	C15—C14—H14	119.9
O1—C7—C8	128.48 (15)	C14—C15—C10	120.24 (17)
N1—C7—C8	105.00 (13)	C14—C15—H15	119.9
C9—C8—C7	101.91 (13)	C10—C15—H15	119.9
			
C7—N1—N2—C9	2.34 (18)	O1—C7—C8—C9	−176.47 (18)
C1—N1—N2—C9	−179.20 (14)	N1—C7—C8—C9	2.23 (16)
C7—N1—C1—C2	−23.6 (3)	N1—N2—C9—C10	177.90 (14)
N2—N1—C1—C2	158.25 (15)	N1—N2—C9—C8	−0.70 (18)
C7—N1—C1—C6	158.12 (17)	C7—C8—C9—N2	−0.98 (18)
N2—N1—C1—C6	−20.0 (2)	C7—C8—C9—C10	−179.50 (15)
C6—C1—C2—C3	−0.9 (3)	N2—C9—C10—C15	−172.87 (15)
N1—C1—C2—C3	−179.19 (15)	C8—C9—C10—C15	5.5 (3)
C1—C2—C3—C4	0.8 (3)	N2—C9—C10—C11	6.0 (2)
C2—C3—C4—C5	0.3 (3)	C8—C9—C10—C11	−175.62 (16)
C3—C4—C5—C6	−1.3 (3)	C15—C10—C11—C12	0.0 (3)
C4—C5—C6—C1	1.1 (3)	C9—C10—C11—C12	−178.90 (16)
C2—C1—C6—C5	0.0 (3)	C10—C11—C12—C13	0.9 (3)
N1—C1—C6—C5	178.26 (15)	C11—C12—C13—C14	−0.7 (3)
N2—N1—C7—O1	175.85 (16)	C12—C13—C14—C15	−0.4 (3)
C1—N1—C7—O1	−2.4 (3)	C13—C14—C15—C10	1.2 (3)
N2—N1—C7—C8	−2.88 (18)	C11—C10—C15—C14	−1.0 (3)
C1—N1—C7—C8	178.87 (15)	C9—C10—C15—C14	177.83 (16)

### Hydrogen-bond geometry (Å, º)

Cg1 is the centroid of the C10–C15 ring.

**Table:**

*D*—H···*A*	*D*—H	H···*A*	*D*···*A*	*D*—H···*A*
C8—H8*A*···O1^i^	0.99	2.36	3.279 (2)	154
C12—H12···N2^ii^	0.95	2.61	3.527 (2)	163
C8—H8*B*···*Cg*1^iii^	0.99	2.69	3.437 (2)	132

Symmetry codes: (i) *x*, −*y*+1/2, *z*+1/2; (ii) *x*, −*y*+3/2, *z*+1/2; (iii) −*x*+1, −*y*+1, −*z*+2.
